# Brief intervention and decrease of alcohol consumption among women: a systematic review

**DOI:** 10.1186/1747-597X-8-31

**Published:** 2013-09-10

**Authors:** Carla Ferreira de Paula Gebara, Fernanda Monteiro de Castro Bhona, Telmo Mota Ronzani, Lelio Moura Lourenço, Ana Regina Noto

**Affiliations:** 1 Department of Psychobiology, Research Center on Health and Substance Use (NEPSIS), Universidade Federal de São Paulo (UNIFESP), Rua Botucatu, 862 – 1° andar, 04023-062 São Paulo - SP, Brazil; 2 Department of Psychology, Center for Studies on Violence and Social Anxiety (NEVAS), Universidade Federal de Juiz de Fora (UFJF), Juiz de Fora - MG, Brazil; 3 Department of Psychology, Center for Research, Intervention and Evaluation for Alcohol & Drugs (CREPEIA), Universidade Federal de Juiz de Fora (UFJF), Juiz de Fora - MG, Brazil

**Keywords:** Brief intervention, Women, Alcohol, Prevention, Systematic review

## Abstract

Problems related to alcohol consumption are priority public health issues worldwide and may compromise women’s health. The early detection of risky alcohol consumption combined with a brief intervention (BI) has shown promising results in prevention for different populations. The aim of this study was to examine data from recent scientific publications on the use of BI toward reducing alcohol consumption among women through a systematic review. Electronic searches were conducted using Web of Science, PubMed(Medline) and PsycInfo databases. In all databases, the term “brief intervention” was associated with the words “alcohol” and “women”, and studies published between the years 2006 and 2011 were selected. Out of the 133 publications found, the 36 scientific articles whose central theme was performing and/or evaluating the effectiveness of BI were included. The full texts were reviewed by content analysis technique. This review identified promising results of BI for women, especially pregnant women and female college students, in different forms of application (face-to-face, by computer or telephone) despite a substantial heterogeneity in the clinical trials analyzed. In primary care, which is a setting involving quite different characteristics, the results among women were rather unclear. In general, the results indicated a decrease in alcohol consumption among women following BI, both in the number of days of consumption and the number of doses, suggesting that the impact on the woman’s reproductive health and the lower social acceptance of female consumption can be aspects favorable for the effectiveness of BI in this population.

## Introduction

Alcohol consumption and alcohol-related problems are considered public health priorities worldwide [1,2]. There are significant gender differences regarding the development of problems caused by alcohol. Compared with men, women’s risk of alcohol use has a disproportionate effect on their lives and health, including consequences on reproductive function and pregnancy [3].

Initiatives aimed at the early detection of risky (hazardous and harmful) drinking have been shown to be effective in preventing alcohol-related social and health consequences [4,5]. Developed for use in primary care, Brief Intervention (BI) has been found to be an effective and low-cost treatment alternative for alcohol use problems. Using self-help strategies, BI aims to promote a decrease in alcohol consumption among nondependent individuals, and in the case of dependence, to facilitate referral to specialized treatment programs [5]. This approach is used in the primary or secondary prevention of alcohol and drug use, which is focused on changing the patient’s behavior through time-limited assistance and is performed by professionals from different backgrounds [4,6]. Given those characteristics, this approach has been considered a relevant practice in the context of public health.

Studies on BIs have demonstrated both its efficacy (ideal world) and effectiveness (real world), in different clinical settings [6-9]. Research suggests that the effectiveness of a BI in reducing problems caused by alcohol can be equal to or even higher than other interventions that require more time to be performed [4,7-9]. There are also different ways of applying this type of intervention, which may vary from counseling sessions with a professional (face-to-face, by phone or online) to self-applied interventions with the aid of manuals or computer-based tools [10-14].

Despite the existence of variations in BI, it is grounded in social-cognitive theory [9] and commonly incorporates some elements of motivational interviewing [15], a behavioral change technique which helps individuals to recognize a problem and motivate them to change it. BI typically provides risky drinkers with feedback on their use, information on the adverse consequences of alcohol; information on the benefits of reducing intake; analysis of high risk situations for drinking and techniques to help moderate their consumption [4,6,9]. The approach also includes an emphasis on the individual’s responsibility for his/her own consumption, empathic attitude, counseling toward changing those behaviors based on the identification of strategies for interrupting or decreasing consumption and stimulus to the patient’s self-efficacy perception. Moreover, the setting of goals to be achieved and reassessed at follow-up sessions is common practice in this type of intervention [4,6,9,16,17].

Data from the WHO (World Health Organization) [18] indicate that alcohol consumption patterns significantly differ between men and women and among age, ethnic, religious and cultural groups, which are key aspects to be addressed in health care. A comprehensive and significant review on BIs in primary care concluded that this approach reduces alcohol consumption in men, but these findings do not extend to women; therefore, research on the most effective components of those interventions in this population is necessary [9]. Thus, the main objective of this study was to examine data from recent scientific publications on the use of BI toward reducing alcohol consumption among women through a systematic literature review.

## Methods

Online searches were performed using Web of Science, PubMed(Medline) and PsycInfo databases. The expression “brief intervention” was linked to the terms “alcohol” and “women” in all three databases, and studies published between 2006 and 2011 were selected.

Out of the 133 papers found, 97 were excluded upon a reading of their abstracts because they were proceeding papers, meetings, book chapters, dissertations, theoretical articles, literature reviews or because they did not address the subject of interest. The papers whose full text was not available in the English language were also disregarded. Most excluded studies consisted of cross-sectional studies that measured the prevalence of alcohol consumption but did not perform a BI, instead suggesting it as a key strategy to be evaluated in future studies. Thus, we included 36 articles that met the following inclusion criteria: a) performed and/or evaluated the effectiveness of a BI; b) performed a BI toward alcohol consumption (not other drugs); c) presented women as part of the studied sample.

The full texts were reviewed through a thematic and structural content analysis, undergoing several stages of data organization [19]. The first stage of analysis was a brief reading for general evaluation. Subsequently, the material exploration began through a vertical analysis of the data, thereby establishing categories and subcategories according to the most common topics found. The following categories of interest to the study were systematized: year and publishing journals, research design, samples with emphasis on women, tools for evaluating alcohol consumption, differentiated measurements of that consumption according to gender, setting where the intervention was performed, duration of sessions and main outcomes of the studies.

Two researchers performed the vertical analysis; however, in cases of disagreement as to the article categorization, a referee with experience in content analysis was defined and asked to reach a final consensus. A comparison and horizontalization of the categorization of each article was performed for a data overview as the final analysis stage. This phase consisted of joining and summing the frequency of categories and subcategories classified in each publication [19].

## Results

Among the 36 articles included, 14 were published in 2006/2007 [16,20-32], 10 in 2008/2009 [8,11-14,33-37] and 12 in 2010/2011 [3,38-47]. The journals that published the most articles were the following: Journal of Studies on Alcohol and Drugs (4) [8,28,36,42] and Alcoholism-Clinical and Experimental Research (4) [12,27,40,47], which were followed by Addictive Behaviors (3) [16,24,43], Journal of Consulting and Clinical Psychology (3) [22,32,37] and Journal of Substance Abuse Treatment (3) [20,30,46]. The vast majority of studies (28) were performed by North American institutions, while 4 studies were performed in Sweden [10,11,29,39], 2 in Australia [14,33], 1 in Switzerland [28] and 1 in Denmark [23]. Only 6 studies were not randomized clinical trials [10,16,25,35,43,44].

The analysis of the samples of the 36 studies (Table 1) was aimed at highlighting the articles that performed BIs strictly on women or having them in majority. College students were the most studied population group (10) [14,16,22,24,32,36-38,41,42], followed by pregnant women (8) [10,13,20,25,26,30,35,45], inpatients (4) [8,28,31,40], primary care patients (3) [23,27,39] and women at reproductive age (3) [21,29,47]. Considering the age of the study cohorts, the majority (16) of the articles presented a mean age under 30 years [12,14,16,22,24,29,35-38,40-43],[45,47], while 7 studies showed averages of 30–40 years [3,11,20,21,23,28,30] and 6 above 40 years [8,31,33,39,44,46]. Seven studies [10,13,25-27,32,34], did not present information on the average age of the sample.

**Table 1:** Population groups studied in the 36 research articles selected for review

	**N of articles**
**Exclusively women**	**15**
Pregnant	8
Reproductive age/infertility evaluation	3
Postpartum	2
Others	2
**Patients (men and women)**	**10**
Inpatients	4
Primary care	3
Others	3
**College students (men and women)**	**10**
**General population (men and women, online)**	**1**
**Total**	**36**

Regarding the intervention setting, most studies (23) were face-to-face. In 10 studies, the BI was performed online through a computer [13,14,25,32,38,41,43,45],[47] and by telephone in 3 [11,27,39]. Furthermore, most interventions reported in the articles were performed in a single session (19) [3,8,10,11,13,16,20-26,30],[31,41,45-47] , with the session length varying between 10 and 30 minutes in 10 studies [8,13,20,23,25,26,30,31],[45,46].

The timeline follow-back (TLFB) was one of the most used tools for measuring alcohol consumption and was adopted in 12 of the empirical studies analyzed [8,22,24,28-30,33,34,36,38],[45,47]. The Alcohol Use Disorders Identification Test (AUDIT) was also applied in 12 studies [3,10,11,14,16,23,29,31],[33,37,43,44]. The T-ACE (Tolerance, Annoyed, Cut Down, Eye-Opener), was used in 7 studies [12,20,21,30,45-47]. The Daily Drinking Questionnaire (DDQ) was used in 6 studies [16,22,32,37,38,41].

Of note, among the studies simultaneously focusing on men and women (21), the use of different measurements to characterize female alcohol consumption was identified in 14 of the studies examined, and there were variations in the distinctions adopted between men and women. Among the 5 studies that adapted the AUDIT score, 1 used 7 [16] and 3 studies used 6 [11,43,44] as the female cut-off point, while maintaining 8 as the male cut-off point. In contrast, the study performed on college students with alcohol consumption problems [37] used 10 as the cut-off point in that tool for both genders.

The differentiation most frequently found in the articles (in 7 of them) refers to the heavy episodic drinking on a single occasion, as follows: 4 or more doses for women and 5 or more doses for men. Regarding a weekly limit, 3 studies considered a female consumption of 7 or more doses for the period, while the male consumption equivalent was 14 or more weekly doses [8,28,38].

Analyzing the outcomes obtained with the BIs it was observed that out of the 36 studies analyzed, 12 of them showed no gender distinctions in the results [14,16,22,24,28,31,36,40-44]. The 24 articles with separate results for women were classified in 3 groups (Tables 2, 3 and 4) according to the following study specificities: the first group comprised the studies whose main aim was to assess the effectiveness of BI in decreasing alcohol consumption; the second group assessed secondary outcomes of BI; and the third group compared different types of interventions.

**Table 2:** Clinical trials assessing the effectiveness of brief intervention in decreasing alcohol consumption with women-specific results

**Authors**	**Study population**	**Type of BI**	**Result**
Chang G, *et al.* (2006) [21]	Women undergoing infertility evaluation	Face-to-face	Neutral
Floyd RL, *et al.* (2007) [29]	Women at reproductive age	Face-to-face	Positive
Delrahim-Howlett K, *et al.* (2011) [47]	Women at reproductive age	Computerized	Neutral
Chang G, *et al.* (2011) [46]	Women with medical diagnostics	Face-to-face	Neutral
O’Connor MJ, *et al.* (2007) [26]	Pregnant women	Face-to-face	Positive
Witbrodt J, *et al.* (2007) [25]	Pregnant women	Computerized	Positive
Tzilos GK, *et al.* (2011) [45]	Pregnant women	Computerized	Positive
Fleming MF, *et al.* (2008) [12]	Postpartum women	Face-to-face	Positive
Begun AL, *et al.* (2011) [3]	Incarcerated Women	Face-to-face	Positive
Beich A, *et al.* (2007) [23]	Primary care patients	Face-to-face	Negative
Brown RL, *et al.* (2007) [27]	Primary care patients	Telephone	Neutral
Lin JC, *et al.* (2010) [39]	Primary care patients	Telephone	Positive
Eberhard S, *et al.* (2009) [11]	Psychiatric outpatients	Telephone	Positive
Saitz R, *et al.* (2009) [8]	Inpatients	Face-to-face	Positive
Larimer ME, *et al.* (2007) [32]	College students	Computerized	Positive
Bingham CR, *et al.* (2010) [38]	College students	Computerized	Positive

**Table 3:** Studies indirectly assessing the impact of brief intervention (face-to-face)

**Authors**	**Population**	**Aim**	**Result**
Chang G, *et al.* (2007) [30]	Pregnant women	To assess the stage of change as a predictor of alcohol consumption	Women in the pre-contemplation or action stages of change reduced alcohol consumption
Chang G, *et al.* (2006) [20]	Pregnant women and their partners	To examine the impact of an alcohol-drinking goal, set during a prenatal BI, on subsequent consumption.	The selection of a goal was highly predictive of subsequent drinking behavior. A prenatal BI significantly reduced alcohol intake, particularly in women with higher levels of earlier consumption. The BI effects were significantly better when a partner participated.
Yonkers KA, *et al.* (2009) [35]	Pregnant women	To describe the adaptation of a behavioral approach to substance use	On average, the days of alcohol and drug use during the previous month decreased by almost half at the endpoint
Fleming MF (2009) [34]	Postpartum women	To assess the effectiveness of a BI on alcohol use in reducing psychological distress.	Significant decrease in depression scores in women who received the BI on alcohol use. There was no significant decrease in depressive symptoms in the control group.

**Table 4:** Studies comparing different types of interventions

**Authors**	**Population**	**Aim**	**Result**
Nilsen P, *et al.* (2010) [10]	Pregnant women	To compare a standard intervention of counseling on alcohol with a comprehensive questionnaire-based counseling model.	An equal proportion of women in both groups stopped drinking during pregnancy, but more women receiving the questionnaire-based counseling said that they had received sufficient information and to a greater extent believed the advice was coherent and easy to understand than the women who received standard treatment.
Armstrong MA, *et al.* (2009) [13]	Pregnant women	To compare two BIs on alcohol use, with one focusing on abstinence and the other geared toward reducing consumption, that were supplemented with a computer-based tool for consumption measurement	There were no significant differences between the results of the 2 intervention groups, but both intervention groups had better outcomes than the control.
Baker AL, *et al.* (2009) [33]	Depressed patients	To compare the effectiveness of a single-focused BI and that of integrated psychological interventions on depression and coexistent alcohol-related problems	In comparison with single-focused interventions, the integrated treatment was linked to a greater reduction in the number of days of consumption and levels of depression. Women showed better results for both variables when subjected to treatment focused on depression than treatment focused on alcohol.
Carey KB, *et al.* (2009) [37]	College students	To evaluate the effectiveness of a motivational face-to-face BI and that of a computer program	Women receiving face-to-face BI reduced their drinking more than women receiving the computer-based intervention

Table 2 shows the 16 clinical trials assessing the effectiveness of a BI in decreasing alcohol consumption, with specific results for women. Among the 9 studies whose sample was exclusively composed of women [3,12,21,25,26,29,45-47], 6 of them obtained positive results [3,12,25,26,29,45] with respect to the expected outcomes, while in 3 [21,46,47], the effectiveness of the BI could not be demonstrated (and the result was classified as neutral). The other 7 studies had samples comprising both genders, and the results found were positive in 5 of them, as follows: 1 study [11] found no significant differences between the genders and was effective for both men and women; and in 4 of them [8,32,38,39], more changes were found in women than in men. Conversely, 2 articles reported better results with a male audience, with the female consumption in 1 study increasing following the BI [23] and the result being classified as negative, while in the other study [27], the effectiveness of the BI was not clear for women.

Table 3 shows four studies [20,30,34,35] which indirectly assessed the impact of the BI on other phenomena in addition to alcohol use (i.e., depressive symptoms and stages of motivation toward change). Although BI has been performed in all these studies, the assessment of its effectiveness was not the main research focus.

Table 4 shows another 4 studies [10,13,33,37] that were aimed at comparing different types of interventions. In all these studies, the BI can be considered effective in decreasing alcohol consumption among women, regardless of the way in which it was performed.

## Discussion

This literature review identified promising results of BI for women, especially pregnant women and female college students, in different forms of application (face-to-face or by computer or telephone) despite a substantial heterogeneity in the clinical trials analyzed. In primary care, which is a setting involving quite different characteristics, the results among women were rather unclear.

It is worth noting that most empirical studies with a sample exclusively composed of women addressed pregnant women, given the impact of alcohol consumption on reproductive issues. In general, these studies indicate that BI was effective in decreasing alcohol consumption among participants of the experimental group. However, the control group participants also tended to reduce their consumption, a finding that coincides with the previous review on BI during pregnancy [48]. It is believed that pregnant women are usually highly motivated toward decreasing alcohol consumption and that the change in context brought by pregnancy provides an opportunity to break the drinking habit [17,48,49]. Therefore, given their condition, obstetric patients would already have a strong motivation to stop or decrease alcohol consumption, favoring the effectiveness of the intervention.

In this sense, studies with pregnant women generally address the incidence of Fetal Alcohol Spectrum Disorder (FASD), which includes a myriad of biological and behavioral disorders and may be regarded as the best-known consequence of alcohol drinking during pregnancy [13,25,26,45]. Because a substantial number of women identify their pregnancy after a few months Floyd *et al*. [17] indicated the need to develop universal strategies aimed at alcohol consumption among women of reproductive age in different contexts and cultures, instead of only among obstetric patients. Such strategies could prevent the alcohol-related harms in the early months of pregnancy, when it is still unknown. Although some studies have indicated that light drinking during pregnancy is not associated to cognitive or behavioral problems in childhood [50-52], it remains unclear what level of alcohol consumption is safe during this phase and how this consumption can affect individual susceptibility [52].

It is worth noting that seven articles focused on non-pregnant female populations [3,12,21,29,34,46,47]. However, each study was performed on women with unusual health conditions and socio-demographic characteristics. There was a decrease of the participants’ consumption in all studies.

With regard to alcohol consumption among college students, it should be noted that this group is in a phase of life with specific characteristics, such as their age and the social activities related to this stage of life, that are associated with excessive drinking. Thus, alcohol use may cease upon graduation or may continue, thereby leading to serious problems. In this context BI is a key prevention strategy [53].

Among the studies analyzed involving college students, all results indicated a decrease in the levels of consumption in both genders [14,16,22,24,32,36-38,41,42], and the studies that showed gender differences pointed to better effects among female participants [32,37,38]. These data on BI with college students, especially the best results on female audiences, were also indicated in a review study on the subject. It highlighted that online interventions have been effective among college students and that women seem more involved in that type of approach, which can be related to the anonymity provided in the face of the stigma regarding alcohol consumption problems in the female population [53].

The context of primary health care, which is considered to be strategic for the detection and prevention of alcohol use as a function of its scale of operation [1], was addressed in only three studies [23,27,39], reaching different results with regard to the female audience. Lin *et al*. [39] found a higher probability of decreasing consumption in Hispanic or non-Caucasian women following a BI by telephone in a sample composed of men and women with a mean age of 68 years. In addition, by applying a BI by telephone, Brown *et al*. [27] obtained a statistically significant decrease in the consumption of men only, while women of the intervention and control groups (predominantly Caucasian and aged between 20 and 39 years) showed a considerable reduction in alcohol consumption, albeit with no significant difference between the female groups. The authors believe that the initial measurement, which involved several questions about alcohol consumption, even addressing aspects of readiness to change that type of behavior, may have influenced the changes observed in the studied women.

In contrast, the study conducted by Beich *et al*. [23] focused on primary health care in Denmark (contrary to previous studies performed in the USA) and observed that women appear to show more defensive reactions than men when faced with a BI and may be more sensitive to criticism of their alcohol consumption. That study found no satisfactory results of a BI application in that context because both genders showed a modest increase of consumption during the follow-up.

The study by Kaner *et al*. [9] emphasized the need to investigate the applicability of BI in the actual context of primary care, considering issues relevant to that setting including the following: unawareness/non-receptiveness of professionals and patients to address issues related to alcohol consumption, time to implement the tool, professional training toward using that approach and the routine of that type of service. The difficulty of implementing a BI in primary care requires that better strategies be identified to adapt practices to the specific circumstances of the context [54].

With regard to the specificities of alcohol consumption in men and women, the WHO [18] indicates that biological factors, including a lower body weight and muscle-fat ratio contribute to making the substance effects appear faster/more intensely in women than in men. Most studies analyzed adopted different criteria to characterize the male and female consumption as risky. However, there seems to be no consensus regarding the quantification of this difference. Most likely, the variation in cut-off points and difference in doses in the tools results from differences in alcohol concentration, which varies from country to country.

Regarding the diversity of tools used for screening, there was a tendency to use more than one tool to characterize alcohol consumption in the samples studied, and the AUDIT and TLFB were the most frequently adopted in the samples analyzed. Beich *et al*. [23] thought that the exclusive use of the AUDIT as a tool to recruit at-risk consumers might not suffice to identify homogeneous samples regarding alcohol use. Thus, future studies involving two or three stages of measurement/identification of consumption patterns may show better results of BI effectiveness. Carey *et al*. [22] observed a decrease in alcohol consumption among college students based on the exclusive application of a TLFB by interview, recommending that the same be used as a BI procedure.

The findings of that study indicating different procedures for implementing BI, including the number and duration of sessions, converge with the summary performed by Nilsen [48]. According to the author, BIs are not homogenous entities but, instead, are a set of practices with common principles. Floyd *et al*. [29] and Kaner *et al.*[9] indicated the need to identify which components of the BI are most effective for women in subsequent studies.

The identification of studies comparing interventions and assessing the impact of a BI on various aspects of alcohol consumption indicates the connection between this problem and other key variables, in addition to the pursuit of improvement of interventions. In this sense, a key aspect found [34], which still requires further research, is the indirect impact of a BI on psychosocial phenomena and health consequences that may be linked to alcohol consumption in women (such as depression and domestic violence, among others), suggesting the expansion of its preventive approach. The use of a BI with a focus on other problems has shown promising results, but the evidence is still lacking [54].

The limitations of this study include the limited addition of recent studies (last five years) and the option for a descriptive review without performing a meta-analysis. Given the clinical, methodological and statistical heterogeneity of the studies, a more qualitative approach was used to compare findings in this systematic review. Moreover, because the studies reviewed were performed in developed countries, there is a need to produce cross-cultural studies and studies in developing countries to address the socio-cultural aspects related to alcohol consumption and the effectiveness of a BI in different contexts.

## Conclusions

The studies assessing the effectiveness of a BI were performed primarily in clinical settings and healthcare services and, in general, reported a decrease in alcohol consumption in the sample studied, both in the number of days of consumption and in the number of doses (or both). Studies on women in community samples in different cultures could represent an alternative to the difficulties of implementation in health services and provide further data on the effectiveness for this population. The literature reports reviewed suggest that the impact on women’s reproductive health and the lower social acceptance of female consumption are aspects favoring the effectiveness of a BI for this audience.

To the extent that preventing female consumption is critical in terms of public health, public policies that encourage the implementation of integrated screening and BI in clinical practice may have a substantial impact on reducing alcohol related harms, both at individual and population levels, especially in developing countries.

## Abbreviations

BI: Brief intervention; WHO: World Health Organization; AUDIT: Alcohol use disorders identification test; TLFB: Time-line followback; DDQ: Daily drinking questionnaire (DDQ); FASD: Fetal alcohol spectrum disorder.

## Competing interests

The authors declare that they have no competing interests.

## Authors’ contributions

CG and FB collected, analyzed, and categorized the data as well as wrote the article; TR served as a consultant for subject- and text-reviewing procedures; LL judged the content analysis and supervised the writing of the text; AN supervised the methodological design and the writing of the text. All authors read and approved the final manuscript.

## Authors’ information

CG is a PhD student from the Department of Psychobiology of Universidade Federal de São Paulo (UNIFESP). FB is a PhD student from the Department of Psychology of Universidade Federal de Juiz de Fora (UFJF). TR and LM are associate professors from the Department of Psychology of UFJF. AN is an associate professor from the Department of Psychobiology of UNIFESP.
